# Magnetic resonance imaging of classified and unclassified Müllerian duct anomalies: Comparison of the American Society for Reproductive Medicine and the European Society of Human Reproduction and Embryology classifications

**DOI:** 10.4102/sajr.v22i1.1259

**Published:** 2018-04-23

**Authors:** Devimeenal Jegannathan, Venkatraman Indiran

**Affiliations:** 1 Department of Radiology, Government Kilpauk Medical College, India; 2 Department of Radiodiagnosis, Sree Balaji Medical College and Hospital, India

## Abstract

Magnetic resonance imaging (MRI), due to its optimal delineation of anatomy, has become the mainstay in imaging for diagnosing Müllerian duct anomalies (MDA). Pelvic MRI is requested for various conditions such as primary amenorrhoea, infertility or poor obstetric history with regard to MDA, as identifying the exact aetiology for these conditions is vital. Knowledge regarding the classification of MDA is important, as the treatment varies with respect to the different classes. As all the lesions do not fit within the classification of the American Society for Reproductive Medicine, a new anatomy-based classification was established by the European Society of Human Reproduction and Embryology and the European Society for Gynecological Endoscopy, to fulfil the needs of experts. We aim to discuss various classes of classified and unclassified MDA with regard to both the above-mentioned classifications and illustrate some of them using various cases based on pelvic MRI studies.

## Introduction

Embryologically, the two-paired Müllerian ducts fuse in the midline in the lower part by the 6th to the 11th week of gestation. Lateral fusion forms the uterus and upper two-thirds of the vagina and the cephalad parts of the separated ducts form the fallopian tubes. Resorption of the midline septum results in a single uterine cavity. The sinovaginal bulb forms the lower third of the vagina, which fuses with the lower part of the fused Müllerian ducts.^1^

Any deviation from the above process leads to Müllerian duct anomalies (MDA). Three-dimensional transvaginal ultrasound is almost as sensitive as magnetic resonance imaging (MRI) in diagnosing congenital uterine anomalies.^2^ However, MRI is preferred, as the T2 sequences enable clear delineation of the uterine zonal anatomy, ovaries, follicles, vaginal continuity to the uterus and vaginal septum.^3^ Though MDA are classified by the American Society for Reproductive Medicine (ASRM) into seven classes, there are some anomalies that are not included in the classification. In order to rectify the deficiencies, a new anatomy-based classification was established by the European Society of Human Reproduction and Embryology (ESHRE) and the European Society for Gynaecological Endoscopy (ESGE),^4^ classifying the anomalies according to the morphology of the uterus, cervix and vagina, independently.^4^

Clinical presentation of the MDA is variable, where patients with obstructive anomalies present earlier. In these young women, the goals of therapy are to relieve the obstruction immediately, restore normal menstruation and sexual function and preserve reproductive potential.^5^ Proper description and classification of the MDA on imaging is vital for planning and instituting appropriate treatment.

## Magnetic resonance imaging sequences

**FIGURE 1:**
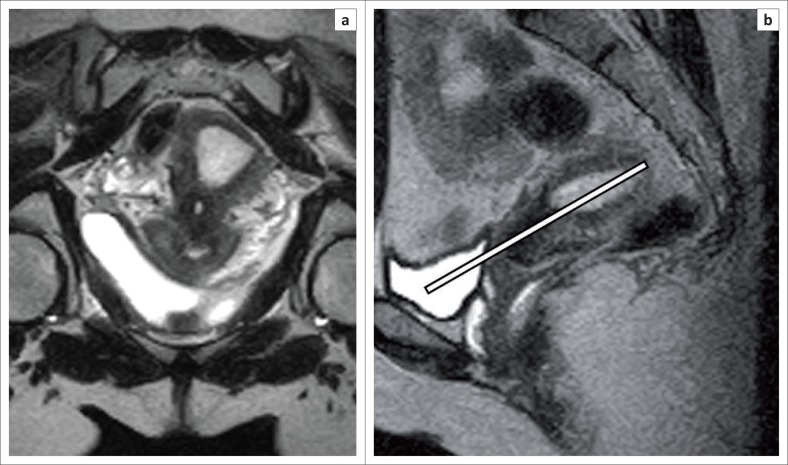
Oblique coronal image (a) obtained by planning along the long axis of uterus (white line) on the sagittal T2 sequence (b).

**FIGURE 2:**
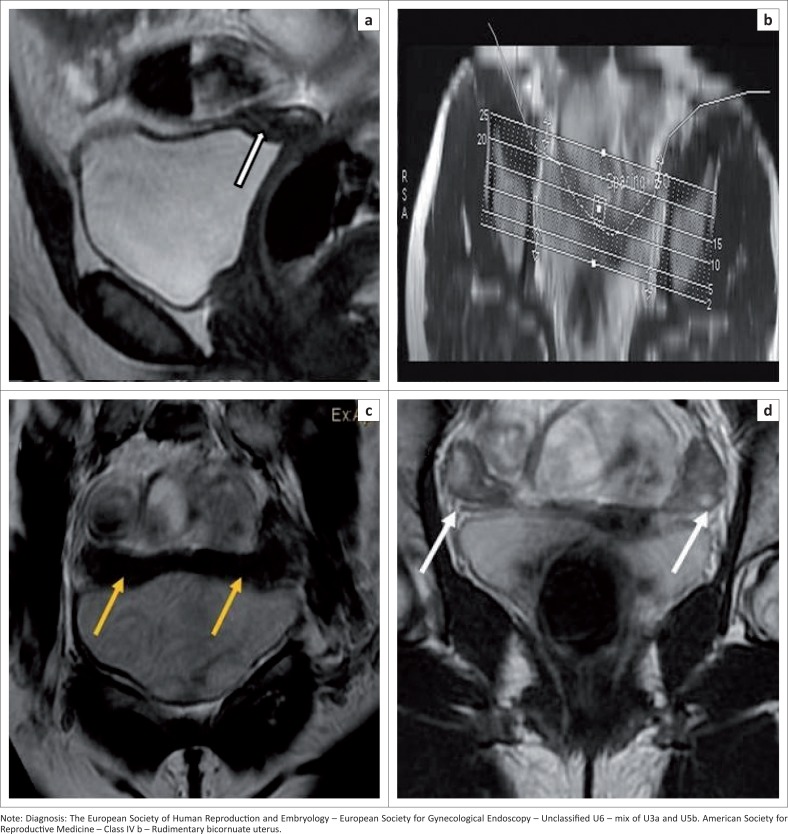
(a) Sagittal T2 sequence demonstrates a rudimentary uterus (white arrow). (b) Planning of the T2 CUBE curved maximum intensity projection (MIP). (c) T2 cube curved MIP demonstrates a bicornuate rudimentary uterus with an intercornual distance of more than 4 cm and widely separated (yellow arrows). (d) Coronal T2 sequence shows both ovaries (white arrows).

T2-weighted sequences in all three planes (axial, coronal and sagittal) are adequate. Firstly, a sagittal T2-weighted image is obtained (TR/TE-4860/86, slice thickness [ST] 5mm and NEX-2), following which oblique coronal (Figure 1) and oblique axial sections are obtained along and perpendicular to the long axis of the uterus. A true coronal section along the long axis of the uterus allows for proper visualisation of the fundal contour. Volume sequences such as T2 CUBE (TR/TE-2000/102, ST2.8 mm and gap 1.4 mm) are very useful in this regard. With this sequence (Figure 2a), reformats in all the planes, including oblique sections, can be determined after image acquisition (Figure 2b). When complex anomalies such as accessory uterus-like structures are seen or in the case of didelphys with a divergent fundus, it is usually necessary to repeat the sequences for each component separately to get the exact coronal and axial sections of uterine cavities. T2 CUBE (GE Medical Corporation) is very beneficial in these circumstances (Figure 2c and d).

**FIGURE 3:**
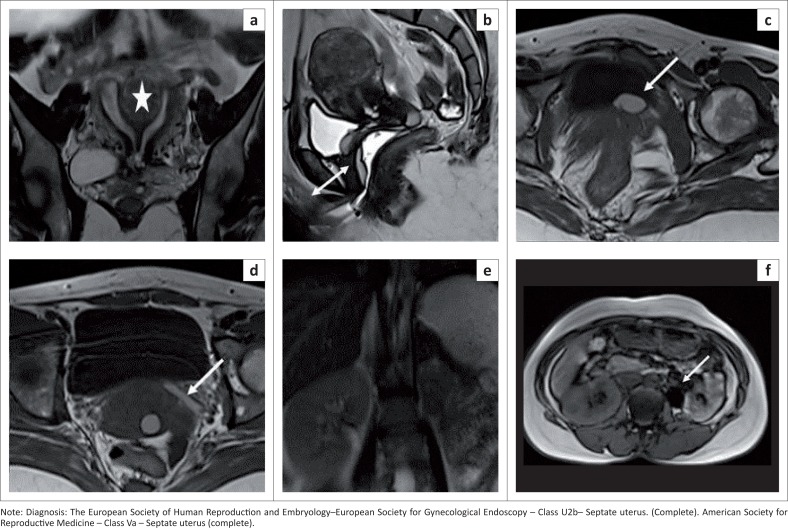
(a) Coronal T2 sequence demonstrates a complete septate uterus with a myoma (star) in the septum. (b) Sagittal T2 sequence shows a Gartner’s duct cyst with focal sacculations (double-headed white arrow) from the fornix extending along the anterior vaginal wall, well delineated against the instilled gel in the vagina. (c and d) Axial T1 sequence demonstrates a left ureterocoele with a blind-ending atretic ureter (white arrows) in the broad ligament. (e) Coronal T2 sequence shows a normal right kidney; left kidney not visualised. (f) Incidental left adrenal adenoma (fat component in out-of-phase images) marked by a white arrow.

T1-weighted sequences are used to define any haemorrhagic component in the obstructed anomaly such as haematometra, adenomyosis, ovarian chocolate cyst and benign teratoma. It is also useful for uterine cysts with mucinous components and paravaginal Gartner’s duct cysts (Figure 3b).

Coronal screening of the upper abdomen is mandatory in all cases of MDA to evaluate for associated renal abnormalities (Figure 3e). Instillation of vaginal gel (Figure 3b) is useful in cases of a blind vagina or transverse or longitudinal vaginal septum. Near-field artefacts can be reduced by placing saturation bands at both the anterior and posterior body walls.

## Classifications

**FIGURE 4:**
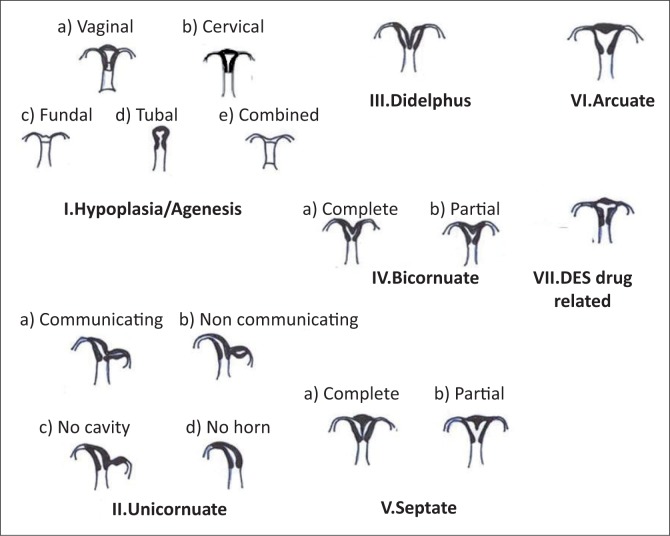
The American Society for Reproductive Medicine classification.

American Society for Reproductive Medicine (Table 1 and Figure 4) and ESHRE and ESGE classifications are used to illustrate the cases here.

**TABLE 1:** American Society for Reproductive Medicine classification.

Class	Classification
Class I	Agenesis or hypoplasia – (a–e) (vaginal or cervical or fundal or tubal or combined)
Class II	Unicornuate – (a–d) (communicating horn or non-communicating horn or no cavity or no horn)
Class III	Uterine didelphys
Class IV	Bicornuate uterus (a and b – Complete or partial)
Class V	Septate uterus (a and b – Complete or partial)
Class VI	Arcuate uterus
Class VII	Diethylstilboestrol related

### The European Society of Human Reproduction and Embryology and the European Society for Gynecological Endoscopy classification

**FIGURE 5:**
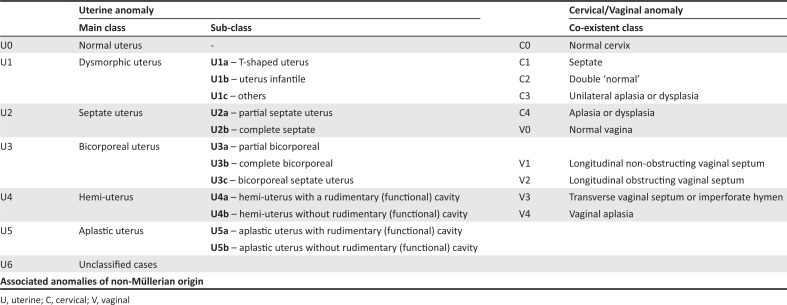
The European Society of Human Reproduction and Embryology and European Society for Gynecological Endoscopy classification.

**FIGURE 6:**
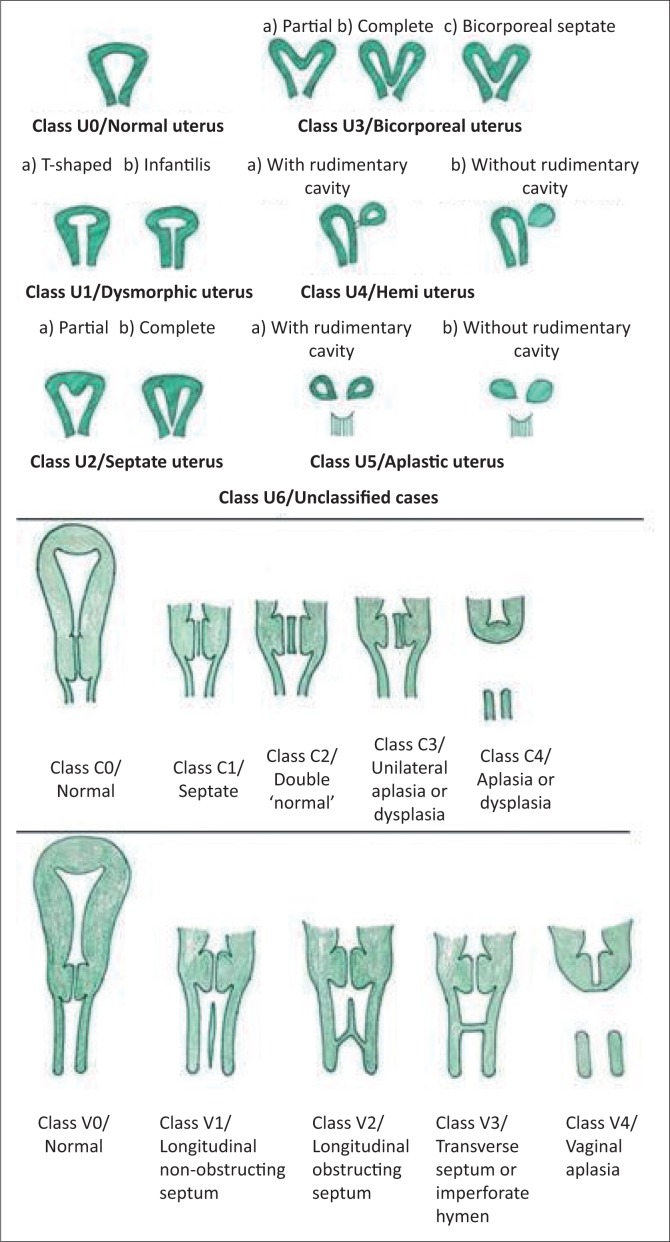
The European Society of Human Reproduction and Embryology and the European Society for Gynecological Endoscopy classification.

Uterine anatomical deviations are classified as normal uterus U0, dysmorphic uterus U1, septate uterus U2, bicorporeal uterus U3, hemi-uterus U4, aplastic uterus U5 and still unclassified cases U6.^4^ Each main class is further divided into sub-classes (Figure 5 and Figure 6).^4^

### The main diagnostic features used in the American Society for Reproductive Medicine classification

1. An external fundal contour with dipping of more than 10% or 1 cm,^6^ indicates a possible diagnosis of uterine didelphys or a bicornuate uterus. Two separate uteri with preserved myo-endometrium zonal widths, two cervices and two vaginas are in keeping with didelphys.^7^ The uterine horns are widely splayed in uterine didelphys. Some degree of fusion in the lower uterine segment points more towards a bicornuate uterus than uterine didelphys.
2. To differentiate bicornuate and septate uterus: an intercornual distance of more than 4 cm, an intercornual angle of more than 105° and external fundal dipping favours a bicornuate uterus.^7,8^
3. Flat or subtle fundal dipping (less than 1 cm), an intercornual distance of less than 4 cm and an acute intercornual angle of less than 75° suggests a septate uterus.^9^ The septum can be muscular or fibrous. A muscular septum is thick and appears similar to myometrial intensity. A fibrous component is hypointense, thin and sharp.
4. In an arcuate uterus, myometrial dipping in the inner aspect of less than 10% is seen with a convex or flat outer fundal contour. The inner dipping edge is not sharp and saddle shaped,^10^ differentiating arcuate from a partial septate uterus.
5. The unicornuate uterus does not have the usual rounded fundal contour and oblong shape. Zonal anatomy and endometrial–myometrial differentiation is maintained.^10,11^
6. Absence of myometrial–endometrial differentiation or fundus, body and cervix differentiation represent a hypoplastic uterus.^8^

### Salient points of the European Society of Human Reproduction and Embryology and the European Society for Gynecological Endoscopy classification

1. Bicornuate and didelphys uterus of the previous ASRM classification incorporate under bicorporeal uterus U3, subcategories 3a and 3b, respectively. A dysfused septate uterus is 3c.
2. Unicornuate uterus is classified under hemi-uterus U4.
3. A clear description of the septate uterus is provided as a fundal midline indentation of >50% of the uterine wall thickness. Anything less is included in the subcategory of U1, a dysmorphic uterus.^4^ The reference for myometrial thickness is obtained on the sagittal image of the uterus by calculating the mean of the measured anterior and posterior wall thicknesses.^12^
4. Arcuate uterus (type VI of ASRM) is not included in the ESHRE–ESGE classification system, avoiding unnecessary apprehension created by this almost normal variant as an anomaly. In the ESHRE–ESGE classification, these patients are classified as normal or septate, depending on the degree of midline indentation.^13^
5. The ESHRE and ESGE classification classifies 38 of the 39 congenital anomalies of the female genital tract, whereas the ASRM classification is not as comprehensive.^13^
6. Independent classification of uterine, cervical and vaginal anomalies is made. It is very useful, especially in cases of obstructive cervical or vaginal malformations with a normal uterus, which constitutes about 22 out of the 39 types of anomalies.^13^ The ASRM classification does not provide specific classes for these anomalies and groups them all under Class I.
7. The ESHRE and ESGE classification uses common terms for describing anomalies and the exact anatomical status of the female genital tract, rather than the liberal non-specific terminologies used in the ASRM classification.
8. The ESHRE and ESGE classification includes associated anomalies of non-Müllerian origin as part of its system to accommodate the renal tract malformations which are common with MDA.
9. The embryological origin of the anomalies can be easily identified on the basis of the ESHRE and ESGE classification.
10. The ESHRE and ESGE classification places less severe anomalies at the beginning with the more deformed anomalies placed as the later classes and sub-classes.^4^
11. The ESHRE and ESGE classification defines uterine deformities based on the proportions of uterine anatomical landmarks, such as uterine wall, rather than using definite objective numerical values, keeping in consideration the variability of uterine dimensions in different patients.^4^

## Ethical consideration

Written informed consent was obtained from all the patients for publication of this review article, including all the images.

## Case reviews

### Normal uterus U0

**FIGURE 7:**
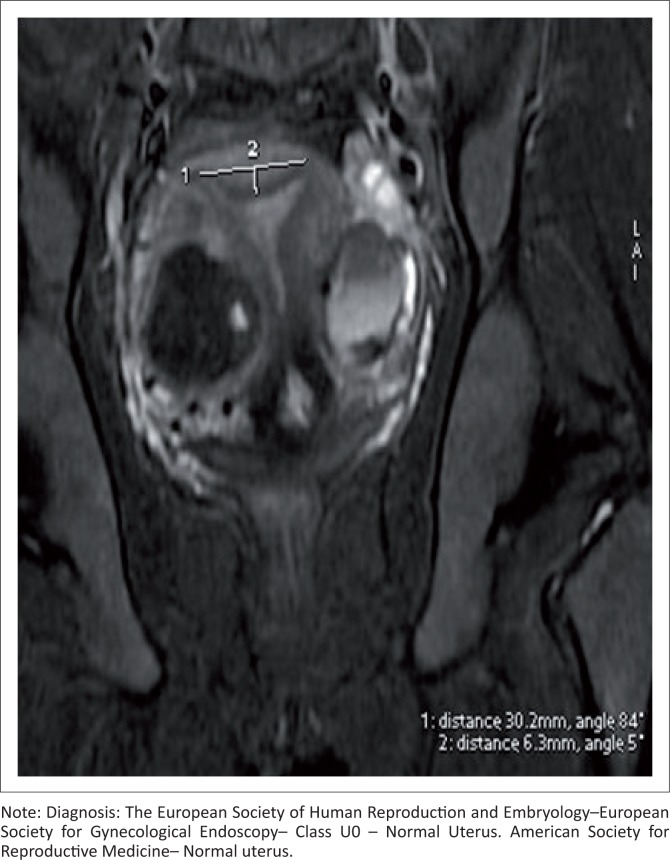
Coronal oblique T2 sequence shows a normal uterus. No fundal external dipping, intercornual distance of less than 4 cm and internal fundal muscular indentation of less than 50% of the uterine wall thickness. Associated right lateral wall intramural fibroid and left benign teratoma seen.

A uterus having an interostial straight or curved line with less than 50% of the uterine wall thickness, internal indentation in the midline fundus and a flat external contour on coronal MRI section is considered a normal uterus U0 (Figure 7).

### Dysmorphic uterus U1

A dysmorphic uterus is sub-classified by the shape of the uterine cavity as T-shaped, infantile and others. The other category is used to differentiate minor deformities.

### Septate uterus U2

**FIGURE 8:**
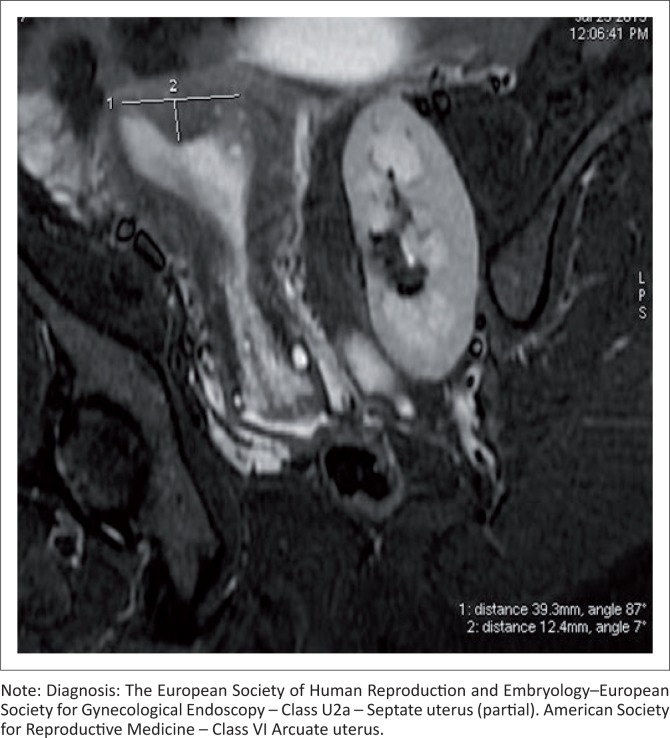
Coronal oblique T2 sequence shows no fundal external dipping, an intercornual distance of less than 4 cm and an internal fundal muscular indentation of more than 50% of the uterine wall thickness. Associated left pelvic kidney is seen.

**FIGURE 9:**
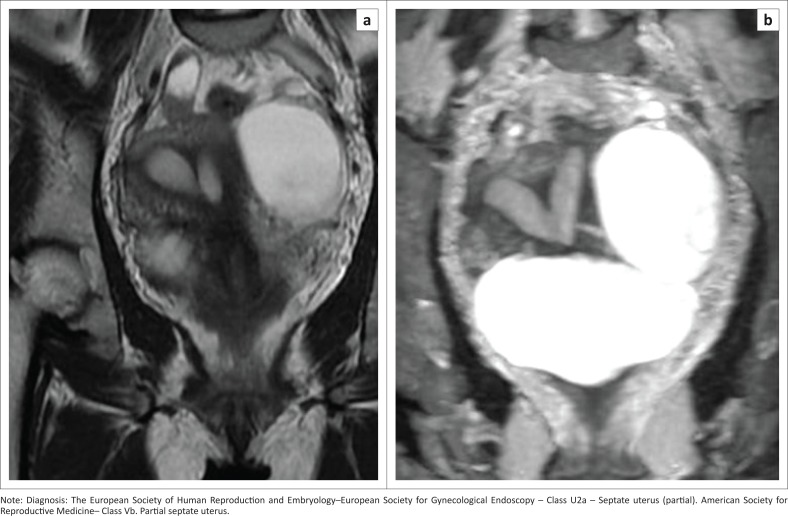
Coronal oblique T2 sequence (a) shows no fundal external dipping. There is a partial septum with a muscular and fibrous component separating the endometrial cavity in the fundal region. A single cervix is seen. T2 CUBE maximum intensity projection (MIP) image (b) demonstrates well the inferior sharp end of the septum. Partial septate uterus. Associated left ovarian cyst.

**FIGURE 10:**
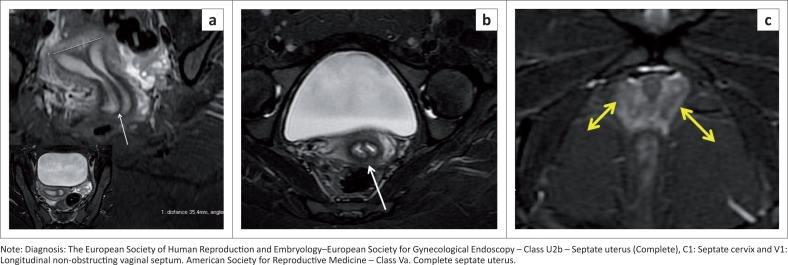
Coronal oblique T2 sequence demonstrates a complete septate uterus. The outer fundal contour is flat. The intercornual distance is 3.5 cm. Internal indentation of the muscular and fibrous component is seen in fundal region (a) and there is only a fibrous component in the lower part (white arrow) (b), extending down to the cervix and two vaginal cavities (c – yellow double-headed arrows).

This is due to an absorption defect. A uterus having a flat external contour and more than 50% internal fundal midline indentation is classified as a septate uterus. It is divided into subtypes a and b (partial [Figures 8 and 9] and complete [Figure 10] septate) by the extension of the septum above or below the internal cervical os, respectively. When two cervices are identified at clinical examination, it should not be interpreted as uterine didelphys, where no treatment is required. Imaging is mandatory.^6^ Septate uteri with two cervices (Figure 10) have been documented in the literature.^14,15,16,17^ The optimum treatment is resection of the septum.^18^

An arcuate uterus may be classified as a partial septate uterus in the ESHRE–ESGE classification (Figure 8). Somayya et al. state that the diagnosis of septate uterus is made frequently by following the ESHRE–ESGE classification, resulting in increased hysteroscopic metroplasty.^19^ This could be considered as one of the limitations of the study.

Ludwin et al.^20^ identified the median length of septum as 10.7 mm by the ESHRE–ESGE classification and as 21.1 mm by the ASRM criteria. In 36.4% of cases of septate uterus, internal fundal indentation was <1 cm by ESHRE–ESGE criteria and is usually considered as a normal uterus by ASRM. Their study further stated that the septate uterus was overdiagnosed and needed to be redefined for determining the treatment option of hysteroscopic metroplasty.^20^ Ludwin et al. stated that using three-dimensional ultrasonography and the ASRM classification with morphometric criteria is preferable and that further study with a larger sample size should be evaluated for reliability.^21^

Patients with a complete septate uterus may have multiple associated findings. Myomas were seen in the septum and left broad ligament in one of our patients (not shown). There was an associated Gartner’s duct cyst that appeared hyperintense on the T1 sequence, extending from the fornix to the anterior vaginal wall. This patient also had a left ureterocoele with a short ureter ending blindly. She had a normal right kidney and an absent left kidney with an incidental left adrenal adenoma (Figures 3a–f). A blind-ending ureteral bud in association with renal agenesis and a ureterocoele are rarely reported.^22,23,24^ Magnetic resonance imaging is the modality of choice for diagnosing an atretic ureterocele; other cross-sectional imaging modalities have limited value.^23^

### Bicorporeal uterus U3

**FIGURE 11:**
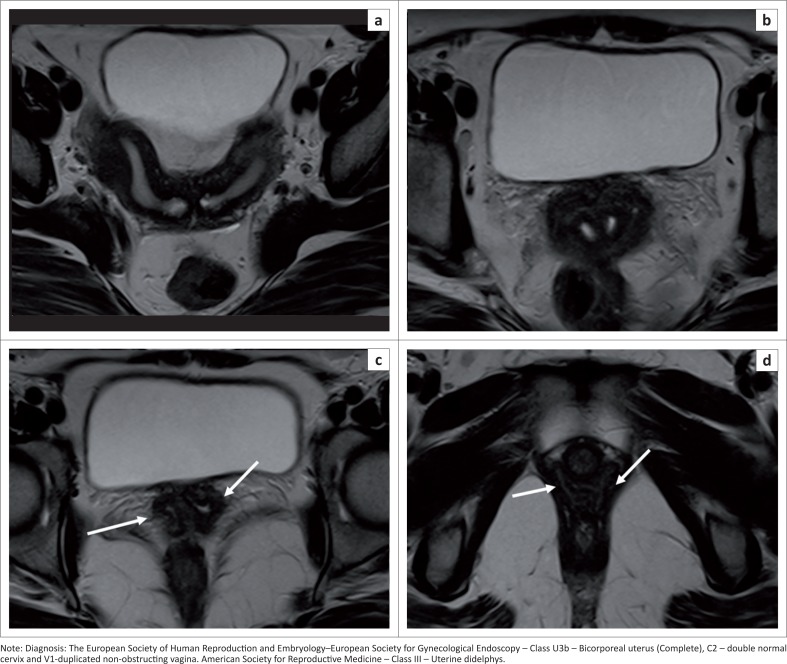
(a, b) Axial T2 sequence demonstrates a fundal external indentation exceeding 50% of the uterine wall thickness, dividing the uterine corpus and cervix completely. (c, d) Axial T2 sequence demonstrates two vaginal cavities (white arrows).

A bicorporeal uterus is caused by fusion defects, characterised by external indentation of more than 50% of the uterine wall thickness. This is subdivided into partial and complete (Figure 11) by the extension of the division above or up to the level of the cervix. A bicorporeal septate uterus (Class U3c) has a coexistent absorption defect in addition to the fusion defect and shows fundal indentation exceeding 150%.

**FIGURE 12:**
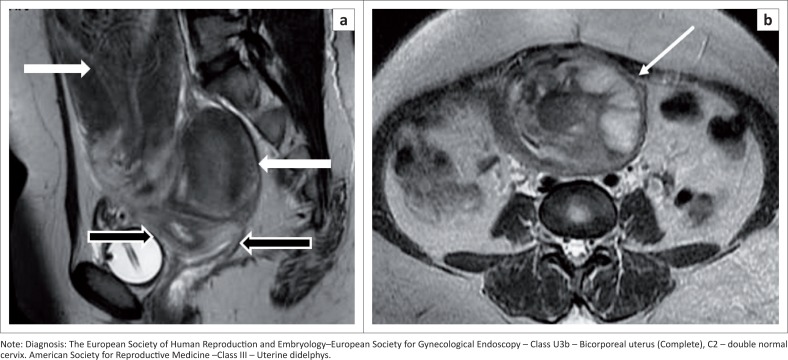
Sagittal T2 sequence (a) demonstrates two fundi and two uterine bodies (white arrows) with two endometrial cavities and two cervices (black arrows). Post-partum changes seen in the anterior uterus. (b) Axial T2 sequence shows post-partum changes with the placenta in fundal and left lateral wall (white arrow) – placenta increta.

**FIGURE 13:**
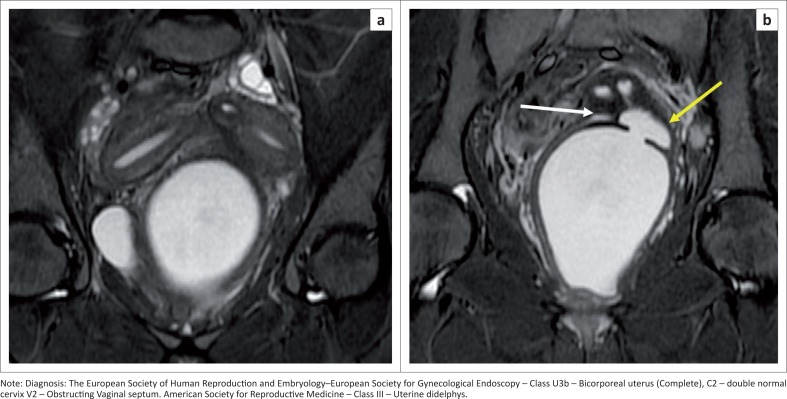
Coronal short tau inversion recovery (STIR**)** sequence (a) demonstrates two uterine bodies with endometrial cavities. Coronal STIR sequence (b) shows two cervices with a compressed right hemivagina (white arrow) and an obstructed fluid distended left hemi-vagina (yellow arrow). It also shows a transverse partial septum. Associated ipsilateral renal agenesis was seen (not shown). Herlyn–Werner–Wunderlich syndrome.

Increased risk of placental abnormalities has been reported with uterine anomalies,^25^ especially with uterus didelphys (Figure 12) and bicornuate, unicornuate and septate uteri. Uterine didelphys (bicorporeal U3b) with a vaginal septum and obstructed hemivagina resulting in haematometra along with ipsilateral renal agenesis is known as Herlyn–Werner–Wunderlich syndrome (Figure 13). The treatment for class U2 and U3c is metroplasty. It is also recommended in cases of arcuate uterus with repeated pregnancy loss.^26,27^

### Hemi-uterus U4

**FIGURE 14:**
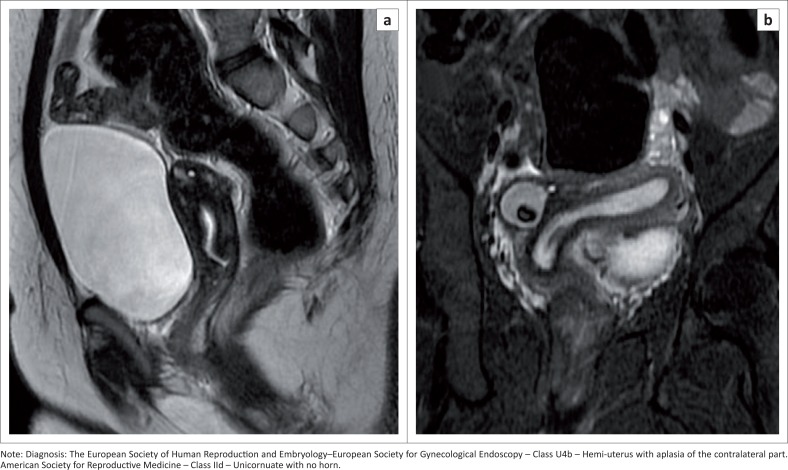
Sagittal T2 sequence (a) demonstrates non-extension of the endometrial cavity up to the fundal region. Coronal oblique T2 sequence (b) shows a unicornuate uterus. Banana-shaped endometrial cavity displaced to left side with right ovarian benign teratoma.

A unilaterally formed uterus, which occurs as a result of a formation defect with an absent or incompletely formed contralateral part, is usually laterally deviated. It is sub-classified into subtypes a and b by the presence or absence (Figure 14) of a functional cavity in the contralateral component (rudimentary horn communicating or non-communicating). Laparoscopic removal of the rudimentary horn is advised if there is a functional cavity. Forty percent of hemi-uteri have associated renal anomalies.^28^

### Aplastic uterus U5

**FIGURE 15:**
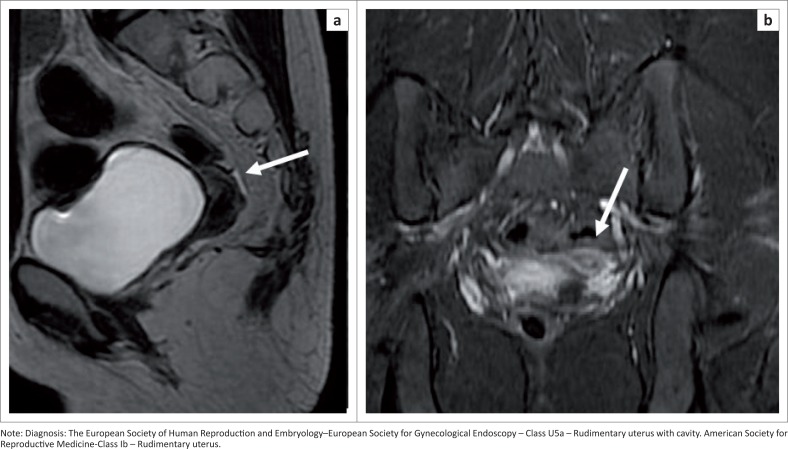
Sagittal T2 sequence (a) demonstrating a rudimentary uterus. Short tau inversion recovery (STIR) coronal (b) shows the rudimentary uterus with a cavity and absent ovaries.

An aplastic uterus is a formation defect with bilateral (Figure 15) or unilateral, complete or partially absent uterine cavity or rudimentary horn, with or without a cavity or uterine remnants. Presence (Figure 15) or absence of a functional cavity sub-classifies an aplastic uterus into subtypes a and b.

Fundal, body and cervix differentiation as well as endometrial–myometrial differentiation are not seen (Figure 2).

**FIGURE 16:**
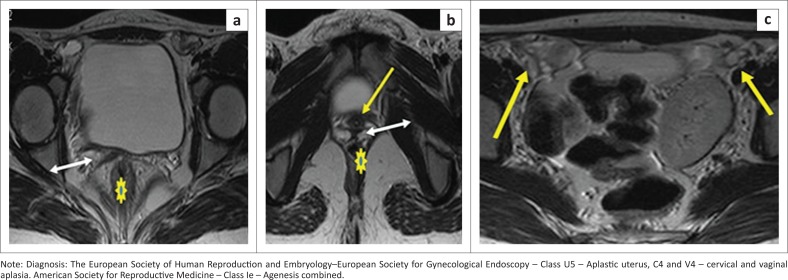
Axial T2 sequence (a) demonstrates a band of vagina (double-headed white arrow) in between the rectum (asterisk) and urethra (yellow arrow). Axial T2 sequence (b) identifies both ovaries (yellow arrows) and a left pelvic kidney. Mayer–Rokintasky–Küster–Hauser syndrome.

Failure of the Müllerian duct to develop leads to Müllerian agenesis. Parikh et al. state that the most common cause of primary amenorrhea is Müllerian agenesis.^5^ Uterovaginal aplasia or hypoplasia is referred to as Mayer–Rokitansky–Küster–Hauser syndrome. In type I or the typical form, there is isolated uterovaginal aplasia. Laparoscopy can identify Müllerian remnants as symmetric muscular buds and normal Fallopian tubes.^29^ In type II (Figure 16) or the atypical form, other upper urinary tract, vertebral, cardiac and otologic malformations are seen.^29^

### Still unclassified cases U6

**FIGURE 17:**
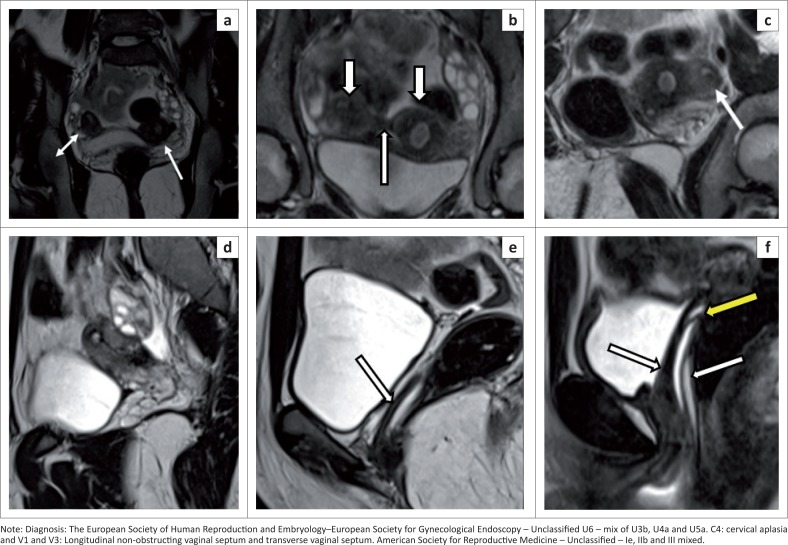
Coronal T2 sequence (a) demonstrates a uterus with endo-myometrial differentiation on the left (white arrow) and an additional uterus-like structure (double-headed arrow) on the right side attached to the left-sided uterus by a fibrous band. (b) Coronal T2 thick maximum intensity projection (MIP) delineates well both uteri (white arrows) and connecting fibrous band (long white arrow). (c) Oblique reformation of the left-sided uterus shows a unicornuate uterus with a rudimentary non-communicating horn (white arrow). (d) Sagittal T2 sequence shows cervical aplasia. (e) Sagittal T2 sequence demonstrates the blind-ended vagina (white arrow). (f) Sagittal T2 sequence with vaginal gel instillation shows a transverse septum superiorly (yellow arrow) and a longitudinal septum (white arrows) in the lower part.

Rare anomalies or combined pathologies that do not comply correctly with one of the above six groups are categorised into unclassified (Figure 17). Ectopic Müllerian tissue anomalies such as an accessory-cavitating uterine mass^30^ and tricavitated uterus^13^ belong to this category.

### Cervical and vaginal anomalies (Figure 6)

**FIGURE 18 (a and b):**
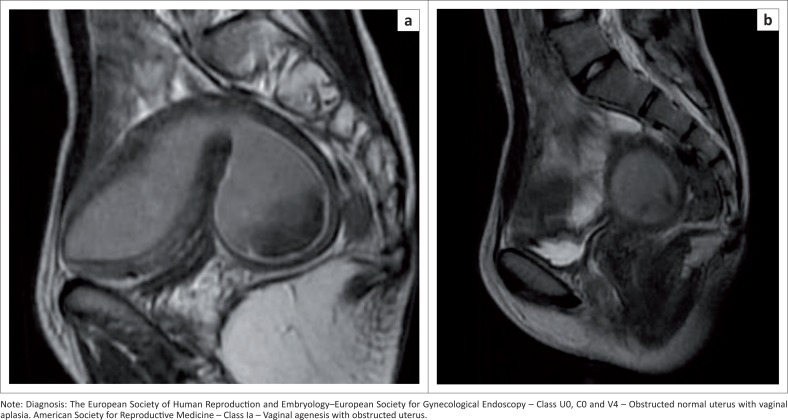
Sagittal T2 sequence demonstrates the uterus and cervix distended with haematometra with a thick non-canalised inferior margin of the cervix.

Figure 18 represents a case of vaginal aplasia (V4).

## Conclusion

The ESHRE and ESGE classification is an anatomical and embryological-based classification and includes cervical and vaginal anomalies. A volumetric T2-weighted MRI sequence such as T2 CUBE is the sequence of choice. Although the ESHRE classification is more accommodative than the ASRM classification, there are still a few unclassified anomalies. Magnetic resonance imaging acquired after the instillation of vaginal gel helps to clearly delineate the transverse and longitudinal vaginal septum. Knowledge of the various classifications, differentiating points, imaging characteristics, associated anomalies, syndromes and various available treatment options is vital for the radiologist to guide the treating experts to achieve the best patient outcome.
